# How to code rare diseases with international terminologies?

**DOI:** 10.1186/1750-1172-9-S1-O11

**Published:** 2014-11-11

**Authors:** Ana Rath, Bertrand Bellet, Annie Olry, Catherine Gonthier, Ségolène Aymé

**Affiliations:** 1 INSERM, US14, Orphanet, Paris, France

## 

Having codes for each and every rare disease (RD) would help European and national health authorities obtain a better knowledge of healthcare pathways and of their impact on specialised health care services (centres of expertise for instance) and on budget. It would also allow having sound data on the epidemiology and natural history of RD, and provide data for clinical research which is critically needed in this field. Improved codification for rare diseases is cited as a priority in the *Council Recommendation on an action in the field of rare diseases* (2009). Currently, only a small fraction of rare diseases have codes in international nomenclatures, making it impossible to trace patients with rare diseases in health information systems on a national and international level. Most European countries use ICD 10 or 9 in their health information systems, some (UK, Belgium, Spain) have decided to adopt SNOMED CT. The number of rare diseases which have a specific code in these two nomenclatures is limited: 466 in ICD10 and 2 883 in SNOMED-CT. In order to provide a common nomenclature specific for rare diseases, Orphanet has established, with the support the European Commission, a nomenclature and classification of RD. The nomenclature and classification are based on scientific publications and on expert advice. It is aligned with every medical terminology in use both in the clinical than in research settings, allowing for interoperability between different coding systems. Terms in the Orphanet nomenclature were introduced in the next ICD11 which is expected by 2017, and will be integrated into SNOMED CT thanks to a partnership between Orphanet and IHTSDO. The Orphanet nomenclature is freely available to download from www.orphadata.org in seven languages (Dutch, English, French, German, Italian, Portuguese, Spanish), and is released twice a year as a PDF document downloadable at http://www.orpha.net[2].

## References

[B1] Rath A Olry A Dhombres F Brandt MM Urbero B Ayme S Representation of rare diseases in health information systems: the Orphanet approach to serve a wide range of end users Hum Mutat 2012 33 5 803 8 10.1002/humu.22078 22422702

[B2] http://www.orpha.net/consor/cgi-bin/Education_Home.php?lng=EN#REPORT_RARE_DISEASES

